# A133 EVALUATION OF DYSPHAGIA IN AN ACADEMIC CENTRE

**DOI:** 10.1093/jcag/gwab049.132

**Published:** 2022-02-21

**Authors:** D E Reed, S Al-Zubide, M J Beyak, W G Paterson

**Affiliations:** Queen’s University, Kingston, ON, Canada

## Abstract

**Background:**

Dysphagia is a common referral to gastroenterology with both benign and malignant etiologies. There is a lack of studies regarding its clinical assessment, complications and outcomes in Canada.

**Aims:**

To evaluate the assessment and outcomes of secondary and tertiary level dysphagia referrals in adult patients.

**Methods:**

We conducted a retrospective analysis of 98 consecutive adult patients who were initially referred for dysphagia to the Division of Gastroenterology at the Kingston Health Sciences Centre in 2015. Chart reviews were performed to assess demographics, characteristics of dysphagia, time for clinical assessment and outcomes.

**Results:**

Of the referred patients, 19 did not present for their clinic appointment. The majority of patients (60%) were >60 yrs old with an equal distribution of male and female patients. In the assessed patients, reflux was the most common etiology (55.5%) followed by oropharyngeal causes (13.5%) and motility disorders (12%). Only 3 patients of the cohort were diagnosed with esophageal cancer, 2 of whom had metastasis at the time of presentation. Time from referral to clinical assessment was <4 weeks in just 30% and *>*12 weeks in 55%. For the patients diagnosed with malignancy, 2 were assessed within of 2 weeks while the third patient was assessed and endoscoped at 12 weeks. In patients sent for endoscopy, 28% had an endoscopy <4 weeks after assessment while 47% had endoscopy *>* 12 weeks after assessment. Manometry was performed in 18% of the patients assessed. Information was available on the course of the dysphagia in 65 patients. Dysphagia had resolved or improved in 83%, remained unchanged in 14% and worsened in 3%.

**Conclusions:**

Reflux-related dysphagia was by far the most common cause of dysphagia in this referral cohort. Wait times for clinical assessment greatly exceeded current CAG recommendations in the majority. Given current resource constraints, this highlights the need for improved referral information and patient care pathways in the management of patients referred with dysphagia.

**Funding Agencies:**

None

